# Stability Evaluation and Design Optimization of Underground Salt Caverns for CAES Under Static and Long-Term Load Conditions—A Case Study of Anning, China

**DOI:** 10.3390/ma19122462

**Published:** 2026-06-09

**Authors:** Hong Ke, Hongling Ma, Yebing Hong, Wenyuan Liu, Zhuo Ma, Longzhen Ren, Xiangqing Li, Jiaqi Yi, Yupeng Yue

**Affiliations:** 1 College for Elite Engineers, China University of Geosciences, Wuhan 430074, China; hke3615@ceec.net.cn; 2 China Energy Engineering Group Yunnan Electric Power Design Institute Co., Ltd., Kunming 650011, China; wyliu4813@ceec.net.cn (W.L.); zma3120@ceec.net.cn (Z.M.); lzren1915@ceec.net.cn (L.R.); xqli101x@ceec.net.cn (X.L.); jqyi3754@ceec.net.cn (J.Y.); ypyue3513@ceec.net.cn (Y.Y.); 3 State Key Laboratory of Geomechanics and Geotechnical Engineering, Institute of Rock and Soil Mechanics, Chinese Academy of Sciences, Wuhan 430071, China; hlma@whrsm.ac.cn

**Keywords:** salt cavern, CAES, multi-cavern injection–production, long-term stability, numerical simulation

## Abstract

At present, research on the long-term stability of multi-cavern coordinated injection–production operations for salt cavern compressed air energy storage (CAES) remains limited. Large-capacity energy storage utilizing multiple interconnected salt caverns has become an inevitable development trend for modern CAES power stations, highlighting the necessity and importance of stability evaluation and design optimization for underground salt cavern storage clusters. Based on the Anning 350 MW CAES demonstration project, this paper takes the abandoned salt caverns of the project as research objects. A three-dimensional geological and cavern model is established using the FLAC3D numerical simulation method, and stability analysis is carried out under static conditions and three long-term gas injection and production scenarios (the pressure conditions are provided by ground-based equipment). The characteristics of the plastic zone, displacement, stress distribution, and volume shrinkage of the caverns are systematically investigated. The results show that under static conditions, the internal pressure significantly controls the development of the plastic zone, and the caverns are generally stable at pressures above 4 MPa. During long-term operation, the plastic zones of each cavern gradually expand, displacements accumulate continuously, and stresses tend to stabilize after an initial accumulation period. After 30 years of operation, no through-going plastic zones appear in any cavern, and all volume shrinkage rates are below 30%. Among the three cases, Case 1 exhibits the best stability, while enhanced monitoring is required for local high-stress regions in Case 3. This study verifies that the salt cavern development for the Anning CAES project is safe and controllable during long-term operation. The layout spacing of caverns is reasonably designed and fully satisfies the stability requirements of salt cavern CAES power stations. The research results can provide a technical guarantee for the construction of the first CAES power station in Yunnan Province and also offer a reliable reference for the design and construction of similar multi-cavity salt cavern CAES projects.

## 1. Introduction

Against the backdrop of global energy transition and the urgent goal of carbon neutrality, large-scale energy storage technology has become the core support for solving the intermittency and volatility of renewable energy (such as wind and solar power), realizing grid peak shaving and valley filling, and constructing a new power system [1,2]. As a major large-scale physical energy storage technology, compressed air energy storage (CAES) has gained worldwide attention owing to its prominent merits, including large installed capacity, long service lifespan, short construction period, environmental benignity and low cost. It has become an indispensable component of the global energy storage industry [3]. Unlike electrochemical energy storage with limited service life and potential environmental risks, CAES relies on the compression and expansion of air to realize energy conversion and storage, which has unique advantages in large-scale long-term energy storage scenarios [4].

The development of CAES technology has gone through decades of evolution, and its technical route and application scenarios have been continuously optimized. Early CAES systems mainly adopted the traditional diabatic mode, which had the problem of low energy conversion efficiency. In recent years, with the breakthrough of adiabatic CAES technology, the energy conversion efficiency has been greatly improved, making CAES more competitive in the large-scale energy storage market [5]. Relevant studies have shown that the energy conversion efficiency of CAES can reach more than 70%, which is significantly higher than that of traditional diabatic CAES [6]. At the same time, the integration of CAES with renewable energy power generation, smart grids, and industrial waste heat recovery has further expanded its application fields and practical value [7].

The performance of CAES systems is closely related to the selection of energy storage media and geological storage bodies. At present, CAES power generation systems can utilize a variety of geological structures, including salt caverns, artificial chambers, abandoned mines, depleted oil and gas reservoirs, and aquifers [8]. Among these storage bodies, salt caverns are recognized as the highest potential and most ideal geological carriers for CAES due to their excellent physical and mechanical properties, such as good airtightness, high bearing capacity, low permeability, and stable chemical properties [9]. Salt caverns are formed by the solution mining of salt mines, and converting abandoned salt caverns into CAES bodies not only realizes the resource utilization of goafs, but also reduces the potential geological risks of salt mines, providing a new path for the sustainable development of the salt industry [10].

Internationally, salt cavern technology for CAES has accumulated mature theoretical foundations and extensive engineering practices. Germany, Canada and other countries have launched a series of research and demonstration projects. As a typical case, Germany’s Huntorf CAES plant—commissioned in 1978—has operated stably with salt cavern reservoirs for over 40 years, well verifying the long-term stability and operational reliability of salt cavern-based CAES [11]. The United States has successively built multiple salt cavern CAES demonstration projects in Texas, Alabama, and other regions, and carried out in-depth research on the stability of salt caverns under long-term injection–production cycles [12]. Canadian scholars have focused on the optimization of salt cavern shape and injection–production parameters, and proposed a set of salt cavern CAES design standards and stability evaluation methods [13]. In addition, scholars from the Netherlands, France, and other European countries have carried out research on the coupling mechanism between salt cavern creep characteristics and CAES operation parameters, providing theoretical support for the safe operation of salt cavern CAES [14].

In terms of theoretical research, scholars have carried out in-depth studies on key technologies such as salt cavern stability, CAES system optimization, and energy conversion efficiency. Some scholars have focused on the long-term creep characteristics of salt rock and the dynamic response of salt caverns under cyclic injection–production conditions, and established a variety of numerical simulation models to predict the long-term stability of salt caverns [15,16]. Scholars have analyzed the plastic zone evolution, displacement change, and stress distribution of salt caverns under different operating pressures, and proposed corresponding stability control measures [17]. In addition, many scholars have combined China’s salt mine geological characteristics, carried out research on the adaptability of salt caverns in different regions to CAES, and optimized the design scheme of salt cavern shape and injection–production parameters [18,19].

However, most existing studies mainly focus on the stability analysis of single salt caverns. Research on the long-term creep behavior, stress coupling effect and plastic zone penetration mechanism of multi-cavern coordinated injection–production operation remains scarce. Moreover, mature systematic evaluation and optimization methods are still lacking. In particular, investigations on multi-cavern stability under the interbedded geological condition of rock-salt–glauberite–mudstone in Anning are still blank, which cannot meet the demands of large-scale development. There are also few directly referable engineering cases and systematic evaluation standards. In view of the above research gaps, it is particularly necessary to carry out targeted research on the operational stability of multi-cavern salt caverns for CAES. Therefore, this study focuses on the planned 350 MW CAES demonstration project in Anning, Kunming. Based on the operational requirements of surface facilities, simulated working conditions are established to reproduce different pressure differences inside salt caverns. The finite difference software FLAC3D is adopted to investigate the stability of salt caverns under static loading and injection–production cyclic conditions. Comprehensive evaluations are conducted on multiple indicators including cavern displacement, stress distribution, plastic zone range, and volume shrinkage rate under various working conditions. This paper aims to establish a feasible scientific evaluation framework and assessment paradigm, provide theoretical support and engineering references for the design, construction and safe operation of subsequent multi-cavity salt cavern CAES projects, and promote the large-scale, high-efficiency and high-quality development of salt cavern CAES technology in China.

In summary, this study innovatively conducts a 30-year cyclic numerical simulation of multi-cavern coordinated injection–production operations in the interbedded rock-salt–glauberite–mudstone formation of Anning, systematically revealing the long-term stability evolution mechanism of salt cavern clusters under static and cyclic loading; it further establishes a feasible stability evaluation framework and optimization paradigm, providing direct theoretical support and engineering references for the design and safe operation of large-scale multi-cavern CAES projects in similar complex layered salt formations.

## 2. Project Overview and Geological Characteristics

### 2.1. Project Overview

The planned Anning 350 MW CAES Demonstration Project aims to utilize the retired salt caverns of Kunming Salt Mine as underground gas storage reservoirs. On the ground, an air compression system, a heat storage and exchange system, and a turbine power generation system will be constructed. The total installed capacity is 350 MW/1750 MWh, with a designed charging duration of 8 h, a rated-power discharging duration of 5 h, and an annual utilization hour of no less than 1500 h.

### 2.2. Geological Characteristics

Kunming salt mine is situated in the northeastern part of the Anning Basin, central Yunnan Province. The Anning Basin is a typical circular structural basin formed under the control of the Indosinian tectonic movement. Salt-bearing strata in the basin are mainly distributed in the Upper Jurassic Anning Formation, which can be divided into three members from bottom to top (J_3_an^1^, J_3_an^2^, J_3_an^3^). The lithological sequence consists successively of gypsum–glauberite, glauberite–halite, and interbedded glauberite–mudstone, forming multiple stable salt ore zones.

At present, exploration in the mining area is mainly concentrated in the Middle Anning Formation underlying the Guogaishan Formation. Four main ore bodies can be identified, which are dominated by interbeds of halite, glauberite and glauberitic mudstone, with interlayers mostly composed of salt-bearing mudstone and glauberitic mudstone.

Latest drilling data show that the total thickness of salt beds in the Middle Anning Formation reaches 350.49 m, which is dominated by interbedded glauberite and halite with a small amount of mudstone. The average NaCl grade of the salt beds is about 60%.

In summary, the salt-bearing beds of the Middle Anning Formation are characterized by moderate burial depth, great thickness, continuous distribution, stable interlayers and high grade, making them an ideal host strata for large-scale salt cavern energy storage reservoirs, and providing a solid geological foundation for well layout design and cavity construction.

## 3. Evaluation Methods of Salt Cavern Stability

In geotechnical engineering practice, FLAC3D offers diverse element types for structural analysis and supports isotropic, transversely isotropic and anisotropic elastic constitutive models. Its plastic models cover commonly used elastoplastic constitutive relations such as the Mohr–Coulomb, Drucker–Prager and Hoek–Brown criteria. Creep behavior can be described by seven built-in creep models including the Maxwell model and power-law model. FLAC3D is capable of solving static, dynamic, creep, seepage, heat conduction and multi-field coupling problems, and is widely applied in complex geotechnical engineering analyses such as salt cavern energy storage, tunnels, slopes, foundations and underground repositories.

Therefore, in this paper, FLAC3D is adopted to analyze and evaluate the stability of salt caverns in the Anning energy storage project under static and long-term loading conditions. The research aims to provide a reference and design optimization basis for the project’s construction, and to offer a referable stability evaluation framework for similar engineering practices.

The basic mechanical governing equation (Cauchy equation of motion) adopted in FLAC3D is given as follows:

(1) $$\sigma_{ij,j}+\rho b_{i}=\rho \frac{d\upsilon_{i}}{dt}$$

where ρ is the material density; $b_{i}$ is the body force per unit element; and $\frac{d\upsilon_{i}}{dt}$ denotes the material derivative of velocity.

The constitutive equation defines the material mechanical behavior and is expressed as:

(2) $${\left[\overset{ˇ}{\sigma }\right]}_{ij}=H_{ij}(\sigma_{ij},\varepsilon_{ij},k)$$

where ${\left[\overset{ˇ}{\sigma }\right]}_{ij}$ denotes the co-rotational stress rate tensor; $H_{ij}$ represents the prescribed constitutive function; and k is a state parameter considering the loading history and process.

The strain-rate tensor is given by:

(3) $$\varepsilon_{i,j}=\frac{1}{2}(\upsilon_{i,j}+\upsilon_{j,i})$$

Based on the above three governing equations, combined with the recommended boundary conditions and initial conditions, a total of fifteen unknowns can be solved, including six components of the stress tensor, six components of the strain-rate tensor, and three components of the velocity vector.

## 4. Stability Evaluation of Salt Cavern Gas Storage Cluster

### 4.1. Methodology

#### 4.1.1. Numerical Calculation Model

**Figure 1:**
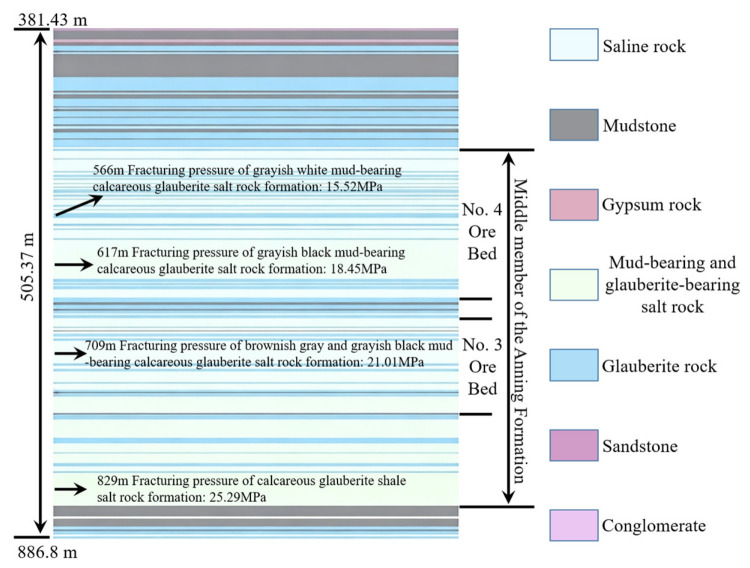
Core column figure.

Based on core sampling data from data wells, representative core column Figure 1 was plotted. In modeling, the core column with the largest coverage of stratigraphic information was adopted to achieve a more comprehensive and detailed description of the stratigraphic structure.

Given the large overall dimension of the numerical model, interlayers and halite beds thinner than 5 m were amalgamated with adjacent strata. This treatment avoids meshing errors and prevents the deterioration of computational accuracy. Meanwhile, to ensure the rationality of boundary settings and the stability of numerical simulation, this study standardized the model boundary as follows: the distance from the model boundary to the cavity edge was uniformly set to five times the maximum cavity radius, so as to eliminate the interference of boundary effects on calculation results.

**Figure 2:**
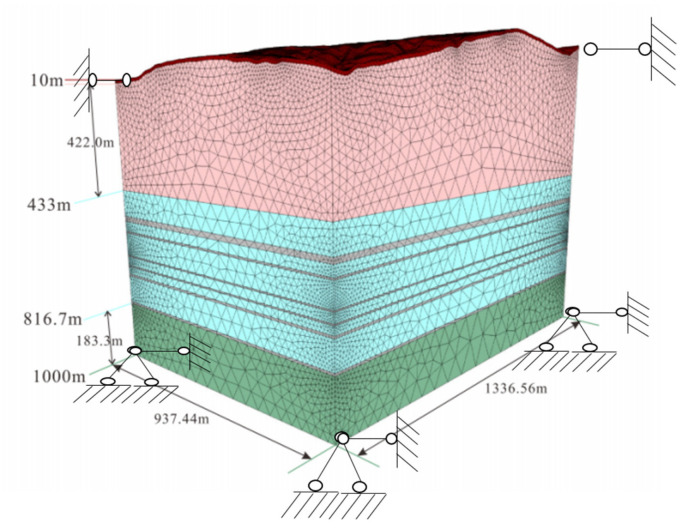
Dimensions of the stratigraphic model.

The maximum cavity radius is determined based on a joint analysis of sonar cavity measurement reports. To avoid boundary interference caused by an excessively small model domain, the maximum cavity radius measured in each of the two surveys for each well is selected as the boundary control parameter. This ensures the model has sufficient extension range and physical rationality to meet the requirements of subsequent stability calculation and response analysis. The dimensions of the stratigraphic model are shown in Figure 2.

**Figure 3:**
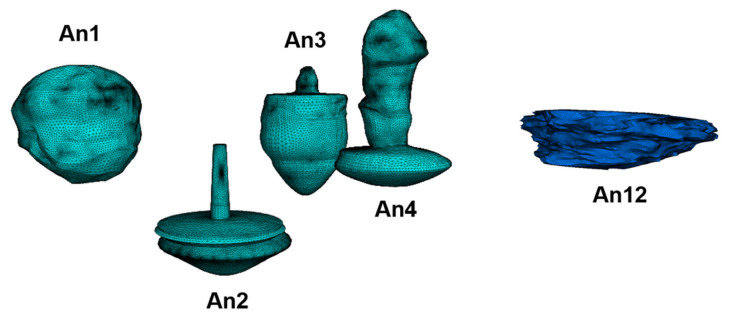
Spatial distribution of cavity model.

The burial depths of the tops of cavities An1 to An4, from shallow to deep, are in the order of An4, An3, An1, and An2. Among them, An1, An2, and An3 are distributed approximately in a straight line. The corresponding spatial–positional relationship is shown in Figure 3.

**Figure 4:**
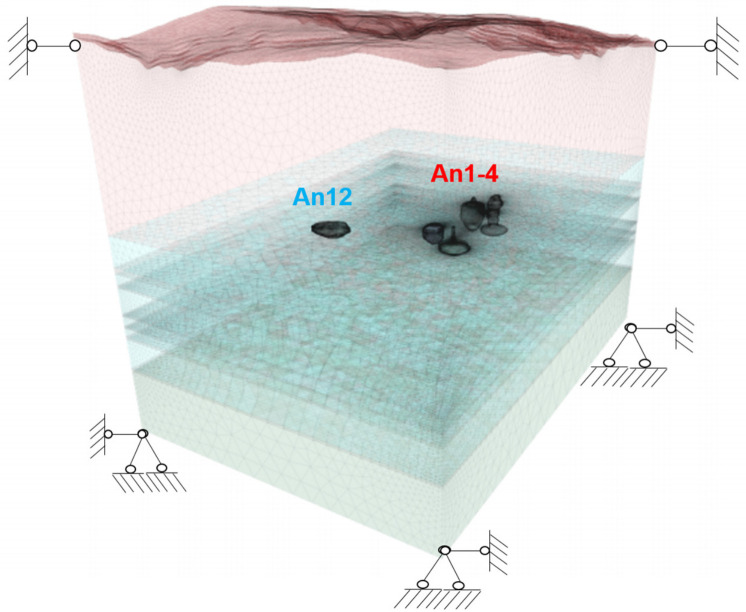
Three-dimensional mesh model.

The established three-dimensional stratigraphic and cavity model was imported into FLAC3D 7.0 for meshing and model initialization. The results are as follows: the number of nodes is 877,084 and the number of elements is 5,131,750. The overall model topology is stable and meets the requirements of subsequent calculation accuracy. The three-dimensional mesh model is shown in Figure 4.

To ensure reasonable boundary conditions and stable simulation results, the following constraints are applied to the model boundaries: The bottom boundary of the model is set as a fixed constraint to restrict vertical displacement. The front, rear, left and right lateral boundaries are applied with simply supported constraints to limit displacement in the normal direction. The geological mass outside the model is regarded as a rigid body without normal deformation. In the numerical model, the salt formation is assumed to be homogeneous, and consistent physical and mechanical parameters are assigned to both salt rock and interlayers. Salt rock exhibits favorable creep characteristics; thus, the in situ stress is considered isotropic and adopted at a gradient of 2.3 MPa per 100 m. The geometries of salt caverns and surface landforms are appropriately simplified and smoothed to avoid computational errors in numerical simulation.

This study aims to provide guidance for the design of future power stations, with the main objective of analyzing the response of salt caverns under different pressure conditions. The FLAC3D software is reliable for calculating salt cavern stability, as validated by the references [20,21,22,23,24]. Accordingly, the numerical results obtained via FLAC3D are fully credible.

#### 4.1.2. Static Stability Evaluation

To evaluate the static stability of the salt cavern gas storage under different operating conditions, static numerical analysis was carried out for cavities An1, An2, An3, An4 and An12. The Mohr–Coulomb elastoplastic model was adopted for the mechanical model of each stratum. This model is a mature and classical elastoplastic model widely used in geotechnical engineering. It can accurately describe the shear-dominated yielding characteristics of rock masses such as salt rock and mudstone, and matches well with the lithological and mechanical properties of rocks in the study area. Meanwhile, its core parameters can be directly measured through core tests with definite physical meanings and high reliability. When applied in FLAC3D, it presents good convergence and high calculation efficiency, which is suitable for large-scale three-dimensional model computation and can fully satisfy the evaluation requirements of plastic zone, stress and deformation.

The Mohr–Coulomb criterion is expressed as:

(4) f=τ−σtanφ−c=0

where *f* is the yield function, *τ* is shear stress, *σ* is normal stress on the failure plane, *c* is cohesion, and *φ* is internal friction angle. All the above parameters are determined by triaxial compression tests.

Expressed in the form of principal stresses, Equation (4) can be rewritten as:

(5) $$f=\frac{\sigma_{1}-\sigma_{3}}{2}\cos\varphi -\left(\frac{\sigma_{1}+\sigma_{3}}{2}-\frac{\sigma_{1}-\sigma_{3}}{2}\sin\varphi \right)\tan\varphi -c$$

where *σ*_1_ and *σ*_3_ are the maximum and minimum principal stresses respectively. Shear failure occurs in salt rock when *f* = 0, and no shear failure happens when *f* < 0.

Tensile failure is generally judged by the criterion that the maximum tensile stress shall not exceed the tensile strength of rock mass, and the formula is as follows:

(6) $$f_{t}=\sigma_{3}-\sigma_{t}$$

where *σ_t_* denotes the tensile strength of salt rock. Tensile failure takes place when *f_t_* = 0, and no tensile failure occurs when *f_t_* < 0.

According to the physical and mechanical tests on cores from data wells, the elastoplastic constitutive parameters of rock salt and interlayers in the Kunming–Anning area are listed in Table 1. The analysis mainly includes two aspects: the overall deformation characteristics of the storage and the stress distribution law around the cavities. To fully reflect the mechanical response of the storage under different internal pressure conditions, several typical static working conditions were set for loading simulation. The internal pressure schemes of the cavities under each working condition are shown in Table 2. The four internal pressure conditions (9.5 MPa, 8 MPa, 6 MPa, 3 MPa and 1 MPa) presented in Section 4.2.3 and the internal pressure values in Table 2 are strictly determined according to the actual design and operation parameters of the 350 MW compressed air energy storage demonstration project in Anning, Kunming. The selection basis is specified as follows:

(1) The upper limit pressure of 9.5 MPa is the rated internal operating pressure of the gas storage cavern, which matches the rated output pressure of the on-ground compressor unit.
(2) The intermediate pressures of 8 MPa and 6 MPa correspond to the regular regulation conditions during gas injection and production, covering the typical pressure range of daily operation cycles.
(3) The lower limit pressures of 3 MPa and 1 MPa are respectively the low-pressure thresholds for emergency operation and routine maintenance of the project. They are adopted to evaluate the stability margin under extremely adverse working conditions. (These two pressures are not recommended for conventional operation; pressurized operation can be adopted when necessary.)

All working conditions are defined in full accordance with practical operation requirements and official design documents. The settings have clear engineering significance and good field applicability, and can fully reveal the stability characteristics of salt caverns under different internal operating pressures.

**Table 1:** Rock mechanical parameters.

Lithology	Elastic Modulus/GPa	Poisson’s Ratio	Cohesion/MPa	Angle of Internal Friction/°	Tensile Strength/MPa
Saline rock	3.66	0.28	11.04	41.34	1.29
Mudstone	11.56	0.26	11.05	34.44	3.66

**Table 2:** Internal pressure values of cavities under different static conditions.

Condition	Internal Pressure Value/MPa
No.1	9.5
No.2	8
No.3	6
No.4	3
No.5	1

### 4.2. Results and Discussion

#### 4.2.1. Plastic Zone Distribution

(1) Distribution law of plastic zones in cavities and strata

**Figure 5:**
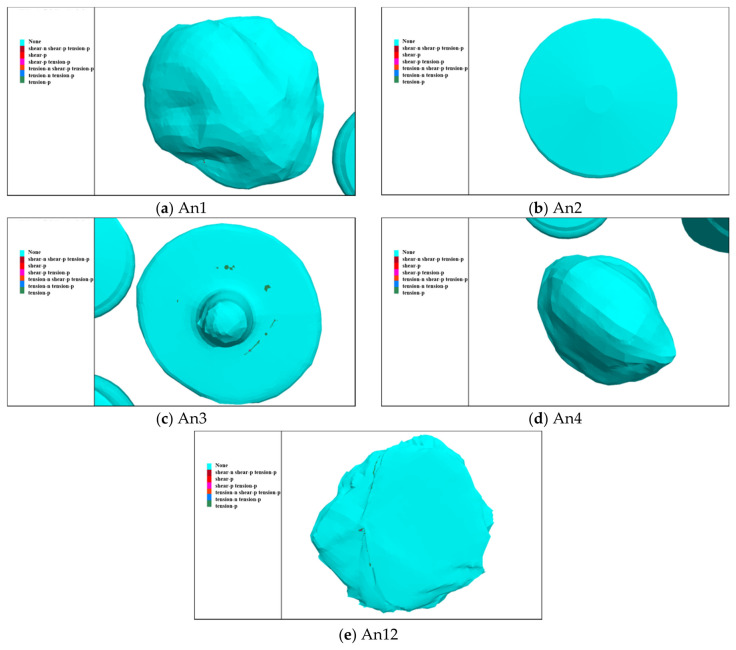
Plastic zone distribution under 8 MPa static pressure.

Taking Condition 2 (internal pressure of 8 MPa) as an example, Figure 5 shows the plastic zone distribution of cavities An1–An4 and An12 under static conditions from a top view. Combined with bottom, left and right views, it comprehensively reflects the stability characteristics of the cavity surrounding rock under static working conditions.

**Figure 6:**
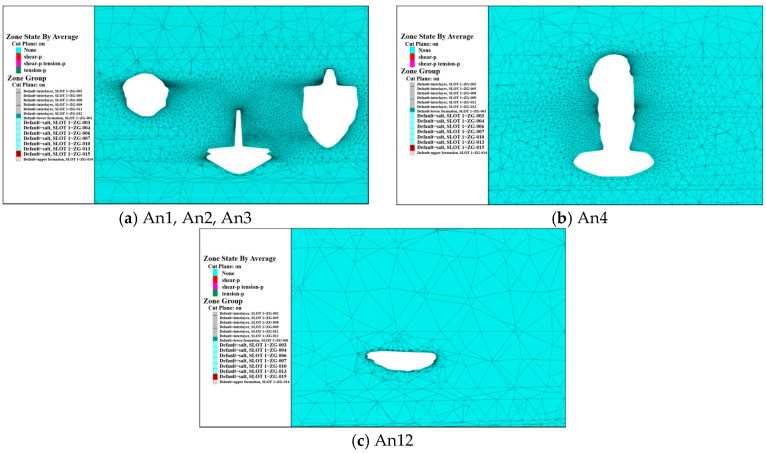
Plastic zone distribution of cavity surrounding rock.

It can be seen from the plastic zone distribution of cavities (Figure 6) that obvious differences exist in plastic zone development among cavities at different well locations: (a) plastic zones in An1 only appear locally and weakly at the edge of the roof, showing good overall stability; (b) plastic zones in an^2^ are mainly concentrated at the bottom and both sides, which is presumed to be related to local interlayers or stress concentration; (c) similar to An1, An3 only shows slight plastic signs in the roof area; (d) no obvious plastic zone is observed in an^4^, indicating the best surrounding rock stability.

Overall, under the static loading condition, the plastic zones around each cavern remain limited in scale and well constrained in extent, without developing continuous failure bands or large-scale instability. At an internal pressure of 8 MPa, no obvious plastic yielding occurs in the cavern surrounding rock and adjacent strata. This demonstrates that the target salt rock formation possesses favorable strength and confining pressure conditions, enabling it to safely withstand the designated operating pressure.

(2) Plastic zone evolution with internal pressure

**Figure 7:**
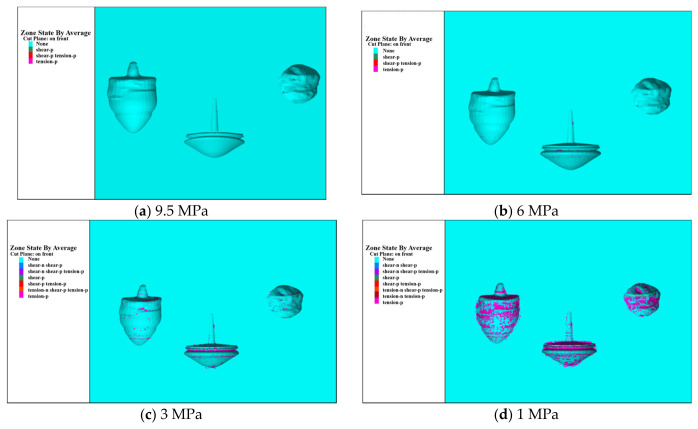
Plastic zone distribution of cavities under different internal pressures.

With the gradual decrease in internal pressure, plastic zones in the surrounding rock of each cavity gradually emerge. The failure mode expands from local to overall, and the failure type also shows a trend from single failure to composite failure. Figure 7 shows the distribution of plastic zones in cavities An1–An3 under varying internal pressures. When the internal pressure is in the range of 3–9.5 MPa, only sporadic plastic zones appear at the interlayer contact zones. When the pressure drops below 3 MPa, plastic zones increase significantly. Tensile failure (tension-p) dominates in the halite layer, while shear failure (shear-p) mainly occurs inside interlayers. Obvious tension–shear composite failure zones (tension-p + shear-p) are distributed on both sides of the interlayers.

In summary, the internal pressure level exerts a strong control on the development of plastic zones. Under high internal pressure, plastic zones are mainly confined to interlayer contact zones. As internal pressure decreases, the scope of plastic failure expands significantly, dominated by tensile failure, and interlayers are often accompanied by composite yielding. Although the evolution of plastic zones differs slightly among individual caverns, all cases follow a consistent pattern: low internal pressure exacerbates rock failure, and interlayers are highly susceptible to tensile–shear composite yielding. This finding provides an important reference for operational pressure regulation and structural safety evaluation of salt caverns.

#### 4.2.2. Displacement Distribution

**Figure 8:**
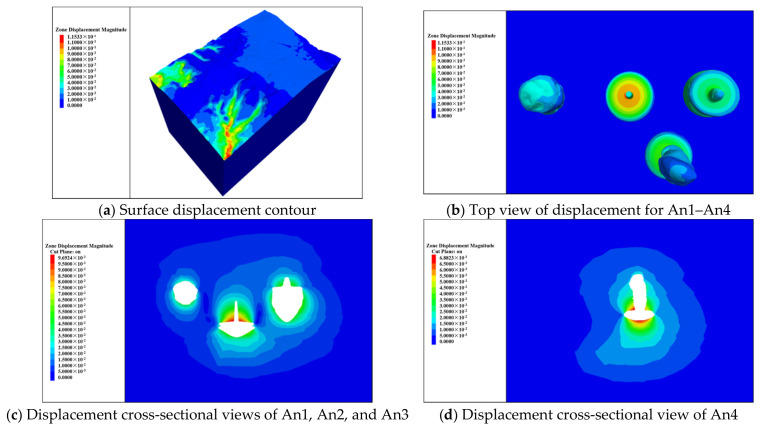
Cavity perimeter displacement distribution.

Taking the internal pressure of 8 MPa as the typical static working condition, combined with the plastic zone distribution around caverns in Figure 6, the plastic zone evolution under different internal pressures in Figure 7 and the displacement nephogram around caverns in Figure 8, this paper systematically conducts coupling analysis on displacement distribution, deformation mechanism, internal pressure sensitivity and stratum response, and fully reveals the displacement evolution law of salt caverns under static conditions.

The total displacement of caverns An1–An4 presents an onion-ring pattern that concentrates at the top and two sides and expands in concentric layers, which gradually decreases outward centered on the cavern without abrupt deformation zones. The surrounding rock is mainly dominated by compression–expansion deformation, and no ultimate penetration failure occurs, indicating favorable overall stability. Obvious deformation differences exist among different caverns. Cavern An2 has the maximum top displacement of approximately 9.7 cm, which is directly attributed to well-developed interlayers at its bottom and sides as well as local stress concentration. Only small-scale concentrated displacement zones appear on the roof of An1 and An3. By contrast, An4 exhibits the minimum displacement with the most uniform distribution and possesses the best stability.

The maximum ground surface displacement reaches about 11.5 cm, and the deformation propagates upward from caverns in a root-like pattern. The cavern deformation is transmitted to the ground surface via weak interlayers, resulting in slight surface uplift. The surface displacement field is continuous and gentle, with no signs of uneven settlement, shear dislocation or collapse. The overall bearing structure of strata remains intact, and the influence of cavern operation on surface buildings and structures is controllable.

Displacement concentration areas are highly consistent with the plastic zones shown in Figure 6. Shear plastic zones synchronously emerge in regions with large displacement at the bottom and two sides of An2, while An4 shows no obvious plastic zones with minimum and uniform displacement. This verifies that plastic development is the core factor inducing displacement increase.

The internal pressure of 4 MPa serves as the critical value for abrupt changes in displacement and plastic zone. When the internal pressure is higher than 4 MPa, the displacement is controllable and the plastic zone development is limited. When it is lower than 4 MPa, both displacement and plastic zone expand rapidly simultaneously, leading to a remarkable decline in static stability.

#### 4.2.3. Stress Distribution Around Cavities

**Figure 9:**
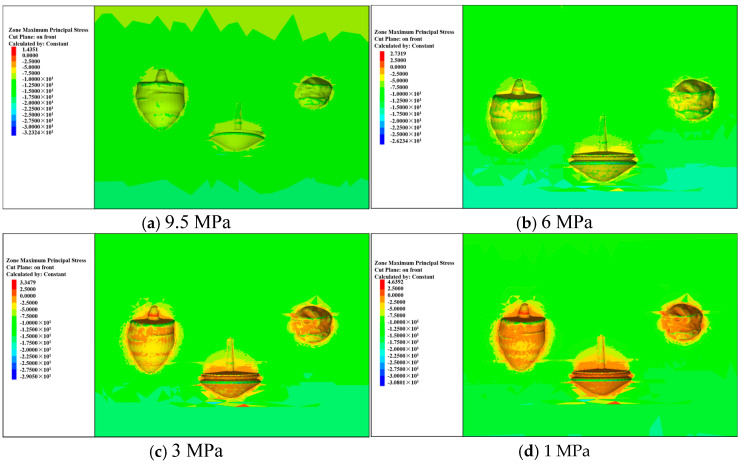
Tensile stress distribution around cavities at various internal pressures.

Figure 9 shows the distribution of the maximum principal stress around cavities An1–An3 under different internal pressures. Generally, as the gas pressure inside the cavities gradually decreases, the surrounding rock transitions from being dominated by compressive stress to locally dominated by tensile stress, and tensile effects gradually appear.

When the internal pressure is in the range of 4–9.5 MPa, cavities An1–An4 are overall under compressive stress, with no obvious tensile zone observed, and the surrounding rock remains in good integrity. When the pressure drops below 4 MPa, the surrounding rock gradually enters a stress conversion stage, and local tensile stress appears near the cavity boundaries. Cavities An1, An3 and An4 first develop tensile stress zones at interlayer boundaries, showing structural-interface-weakening responses. In addition to interlayer positions, cavity An2 also shows local tensile stress concentration at geometric corners, accompanied by strong compressive stress accumulation at the lower interlayer, indicating complex stress reversal at this location. In addition, a certain degree of horizontal tensile trend can be observed in the interlayer strata, which may induce risks such as stratification or bedding dislocation.

### 4.3. Long-Term Stability Evaluation

To evaluate the stability of the salt cavern CAES system under long-term injection–production operation, numerical simulations for long-term stability were carried out on the caverns. A Power-Mohr viscoelastic–plastic creep model was adopted for rock salt and interlayers, while the Mohr–Coulomb elastoplastic model was used for overlying strata, underlying strata and surface soil layers. The cavity region was set as a Null model to represent the energy storage space.

The Power-Mohr creep model follows the Mohr–Coulomb law for both strength criterion and flow rule. Similarly to most existing creep constitutive models, although the specific forms of constitutive equations differ, their physical essence is essentially identical [25,26]. In the steady-state creep stage, the constitutive equation of creep strain rate can be expressed as:

(7) $$\dot{\varepsilon }(t)=Aq^{n}$$

where *q* is the von Mises equivalent stress, defined as $q=\sqrt{3J_{2}}$, and *J*_2_ is the second in variant of the deviatoric stress tensor; *A* and *n* are material constants determined from laboratory tests of salt rock.

Taking the logarithm on both sides of the equation yields:

(8) $$ln\dot{\varepsilon }(t)=lnA+nlnq$$

This relationship can be fitted as a straight line, in which the slope is equal to *n* and the intercept on the vertical axis is ln*A*.

The parameters used are listed in Table 3.

**Figure 10:**
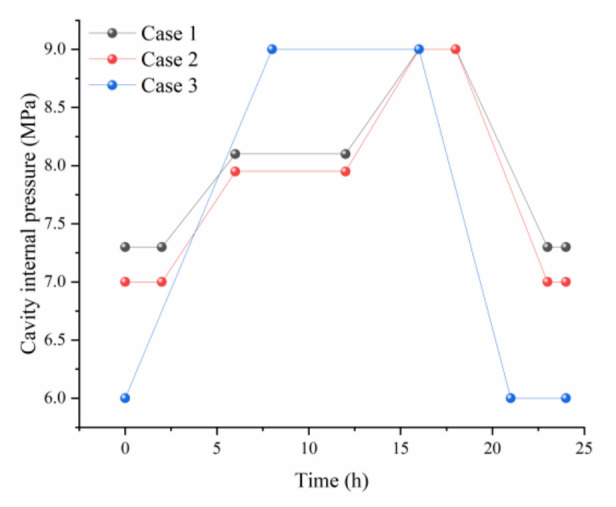
Injection–production operating cases.

To evaluate the stability of salt cavern CAES systems under long-term injection and production operation conditions, three representative operating cases are selected for numerical simulation research. All three cases adopt a 365-day daily cycling mode, in which one complete injection–production cycle is performed each day, with continuous operation for 30 years. During the numerical simulation, different pressure control strategies were implemented to systematically investigate the evolutionary characteristics of plastic zone propagation, displacement accumulation, and stress disturbance responses in the cavern surrounding rock under cyclic injection–production conditions. This analysis comprehensively reveals the stability evolution trend of the salt cavern structure throughout long-term service. The injection–production operating conditions are illustrated in Figure 10.

(1) Case 1: The design operating pressure range for this case is 7.3–9.0 MPa, with two daily charging cycles and one discharging cycle. The specific time and pressure control are organized as follows: ① First charging phase: 02:00–06:00; pressure increases from 7.30 MPa to 8.12 MPa, lasting 4 h. ② Gas storage Phase I: 06:00–12:00; pressure is maintained at 8.12 MPa for 6 h under constant pressure. ③ Second charging phase: 12:00–16:00; pressure rises further from 8.12 MPa to 9.00 MPa, lasting 4 h. ④ Gas storage Phase II: 16:00–18:00; pressure is maintained at 9.00 MPa for 2 h under constant pressure. ⑤ Discharging phase: 18:00–23:00; pressure decreases from 9.00 MPa to 7.30 MPa, lasting 5 h. ⑥ Gas storage Phase III: 23:00–02:00; pressure is maintained at 7.30 MPa for 3 h under constant pressure.
(2) Case 2: The operating pressure range for this case is 7.0–9.0 MPa. The operational rhythm is as follows: ① First charging phase: 02:00–06:00; pressure increases from 7.00 MPa to 7.96 MPa, lasting 4 h. ② Gas storage Phase I: 06:00–12:00; pressure is maintained at 7.96 MPa for 6 h under constant pressure. ③ Second charging phase: 12:00–16:00; pressure rises further from 7.96 MPa to 9.00 MPa, lasting 4 h. ④ Gas Storage Phase II: 16:00–18:00, with pressure maintained at 9.00 MPa for 2 h under constant pressure. ⑤ Discharge Phase: 18:00–23:00, with pressure decreasing from 9.00 MPa to 7.00 MPa for 5 h. ⑥ Gas Storage Phase III: 23:00–02:00, with pressure maintained at 7.00 MPa for 3 h under constant pressure.
(3) Case 3: This case adopts a conventional gas injection and production mode with an 8 h charging and 5 h discharging cycle. The operating pressure range is 6.0–9.0 MPa. The operating rhythm is as follows: ① Injection Phase: 00:00–08:00, with pressure increasing from 6.00 MPa to 9.00 MPa for 8 h. ② Gas Storage Phase I: 08:00–16:00, with pressure maintained at 9.00 MPa for 8 h under constant pressure. ③ Discharge Phase: 16:00–21:00, with pressure decreasing from 9.00 MPa to 6.00 MPa for 5 h. ④ Gas Storage Phase II: 21:00–24:00, with pressure maintained at 6.00 MPa for 3 h under constant pressure.

#### 4.3.1. Plastic Zone Distribution Characteristics

To study the surrounding rock failure characteristics of the energy storage reservoir under long-term operating conditions, the distribution of the plastic zone was analyzed under different operating pressure schemes.

Under the operating schemes of Case 1 and Case 2, the four caverns (An1, An2, An3, and An4) showed significant common characteristics in the distribution and development law of the plastic zone during long-term operation. Overall, they all followed the core law that “with the extension of operating time, the plastic zone gradually expands from local concentration to the whole domain and evolves from scattered distribution to large-scale distribution”. Compared with Case 1, Case 2 develops a small number of newly scattered plastic points around the caverns, while the overall difference remains insignificant. No extensive continuous or through-going plastic zones are generated, and the surrounding rock structure of the caverns maintains satisfactory stability.

**Figure 11:**
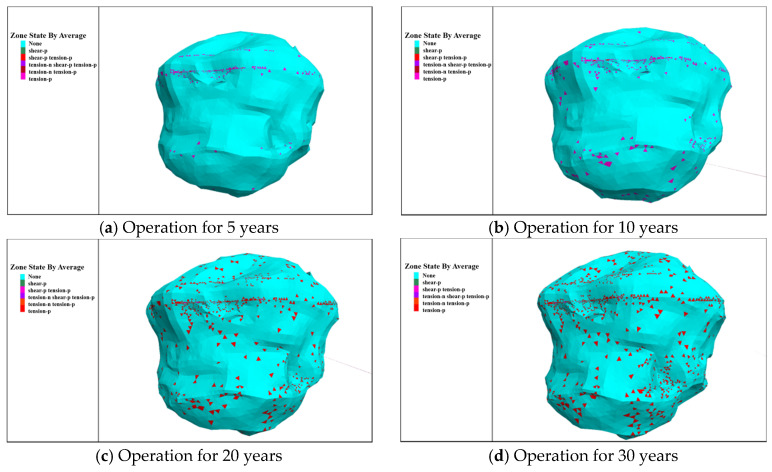
Plastic zone evolution distribution of An1 cavern in Case 1.

In the initial operation period (the first 5–10 years), the plastic zone was concentrated at the contact parts between interlayers and salt rock as well as local areas of the caverns, while only scattered or no obvious plasticity was observed in other areas. In the middle and later periods (10–30 years), the plastic zone gradually expanded and extended to the main salt rock of the cavern body. The main area was dominated by tensile plasticity, and only local shear plasticity appeared on both sides of the interlayers of some caverns. The plastic development in the upper parts of An2 and An3 was not obvious, which was related to their smaller upper diameter and was a common influencing factor. There were also slight differences in the distribution of the plastic zone among the caverns: in An1, only weak plasticity existed at the contact parts of the interlayers in the initial stage, and local shear plasticity appeared on both sides of the interlayers in the later stage; in an^2^, the plasticity was concentrated on both sides and turning points of the lower interlayer in the initial stage, and there was no obvious plasticity in the upper interlayer in the later stage; in an^3^, the plasticity of the upper interlayer was more than that of the lower interlayer in the initial stage, and expanded to the cavern body in the later stage; in An4, the plasticity of the lower part was more than that of the upper part in the initial stage, and the plasticity of the upper part increased slightly in the later stage but was still dominated by the lower part. Due to space limitations, Figure 11 shows the distribution of the plastic zone around the An1 cavern under Case 1.

**Figure 12:**
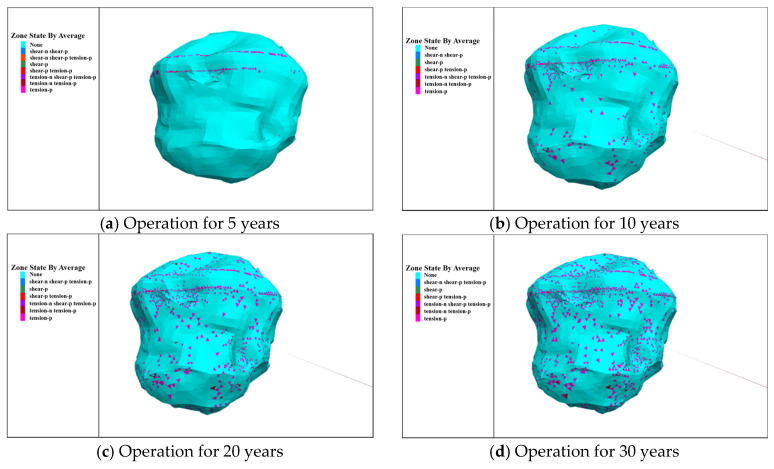
Plastic zone evolution distribution of An1 cavern in Case 3.

As shown in Figure 12, under Case 3, the overall distribution characteristics of the plastic zone remain basically consistent with those under Case 1 and Case 2. However, in Case 3, the number of plastic zones around the cavern increases slightly further, with several scattered plastic points newly formed in local areas, indicating that the local failure trend of the surrounding rock is slightly enhanced under this operating condition.

In summary, after 30 years of operation, no through-going plastic zone has formed around any of the caverns under the three operating cases. Plastic development is mainly scattered, with only relatively continuous plastic zones existing on both sides of the interlayers. Overall, the surrounding rock structure maintains good stability under long-term injection–production conditions.

#### 4.3.2. Displacement Distribution Characteristics

Displacement analysis includes two dimensions: Z-direction displacement and total displacement. Z-direction displacement represents the vertical component of rock mass displacement, which can be used to judge whether stratum settlement (negative value) or uplift (positive value) occurs, so as to evaluate the possible surface settlement and deformation trend of the cavern roof during gas injection and production. Total displacement is the vector norm of displacements of rock mass zones in 3D space (X, Y, and Z directions), reflecting the overall deformation intensity. It can be used to identify displacement concentration zones and assist in determining potential unstable regions of the structure.

**Figure 13:**
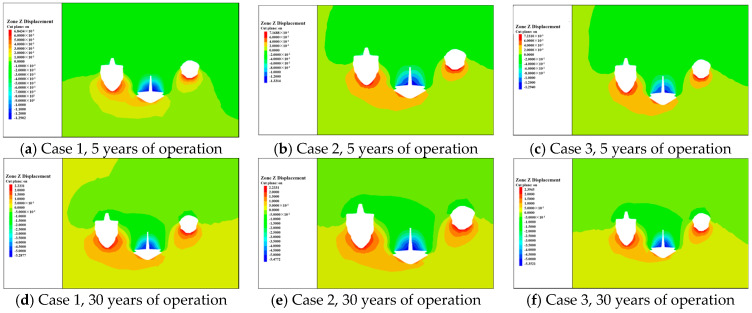
Long-term operation displacement contours of caverns under different cases.

Figure 13 shows the displacement nephograms of the long-term operational stability of caverns An1–An3 under Cases 1, 2 and 3. Under all operating conditions, the caverns exhibit a common deformation trend: upward uplift at the bottom and downward settlement at the roof. Displacement accumulates with operating time, and the overall deformation degree gradually increases. Compared with Case 1, Case 2 yields a slightly larger initial deformation rate and a marginally broader scope of bottom heave and roof subsidence. Nevertheless, the displacement evolution of the two cases gradually converges in the later operation stage, and their deformation discrepancy gradually diminishes. Relative to Case 2, Case 3 exhibits an overall similar displacement distribution pattern, with only a slight increase in the extent and magnitude of cavern uplift and subsidence, suggesting an insignificant overall difference among the three scenarios.

In summary, although slight differences exist in the initial disturbance under various conditions, the structural deformation is well controlled, no significant change occurs in the surrounding rock structure, and the overall stability of the caverns is favorable, with displacements all within an acceptable range.

#### 4.3.3. Stress Distribution Characteristics Around Caverns

To reveal the stress response characteristics of the surrounding rock of salt caverns under long-term cyclic injection–production, the principal stress cloud maps after 30 years of operation were extracted to analyze the three-dimensional distribution of principal stress. This index characterizes the magnitude and orientation of the maximum principal stress in the rock mass, which can effectively identify potential tensile cracking or crushing zones, and is of great significance for evaluating the structural stability of the cavern boundary and interlayer regions.

**Figure 14:**
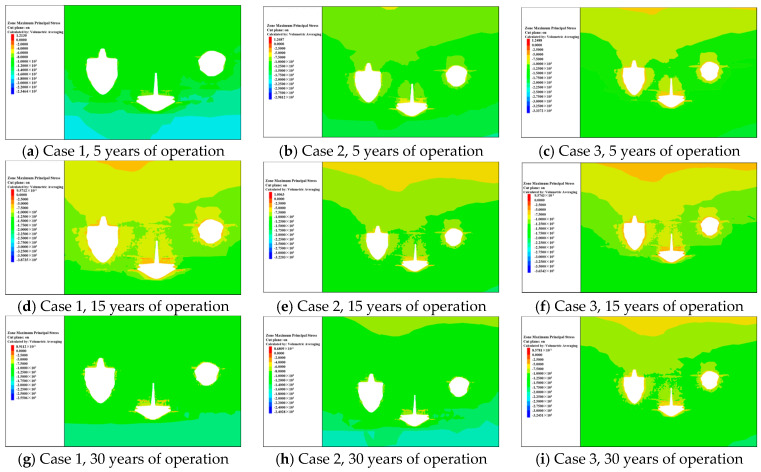
Long-term operation stress contours of caverns under different cases.

Figure 14 shows the nephograms of the maximum principal stress distribution of An1–An3 under long-term operation for Cases 1, 2, and 3. The caverns under all three cases exhibit obvious common characteristics: stress concentration occurs at the cavern boundaries, with an evident tensile trend at the roof and upper edge—whose range is larger than that at the bottom. The stress evolution follows a pattern of gradual accumulation in the early stage (first 20 years) and stabilization or stress release in the later stage (around 30 years). The principal stresses in the surrounding rock of all caverns do not exceed the upper tensile strength limit and remain within a safe and controllable range.

After 30 years of operation, local zones on both sides of the interlayers in An1, An2, and An3 show a transition between compressive and tensile stresses, while the stress distribution of An4 remains stable throughout. Compared with Case 1, Case 2 has a wider influence range of principal stress and a more pronounced tensile trend. Compared with the former two cases, Case 3 shows an even larger stress influence range, more significant expansion of high-stress zones, and delayed stress release at 30 years.

Overall, the stress field distribution is consistent under the three cases. No continuous tensile fracture zones exist in the cavern structures, and no obvious stress coupling zones appear between An1, An2, and An3, indicating that the cavern spacing is reasonably arranged and engineering-feasible. Only Case 3 requires enhanced monitoring of local high-stress regions.

#### 4.3.4. Cavern Volume Shrinkage Characteristics

**Figure 15:**
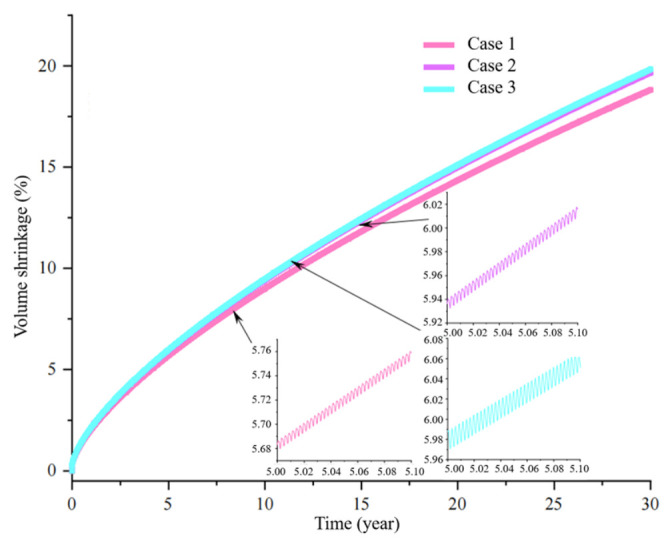
Comparison of cavern volume shrinkage rates under different cases.

Figure 15 illustrates the volume shrinkage of caverns An1–An4 after 30 years of operation under three pressure schemes: 7.3–9.0 MPa (Case 1), 7.0–9.0 MPa (Case 2), and 6.0–9.0 MPa (Case 3). The volumetric shrinkage of the caverns exhibits periodic fluctuations corresponding to the operating pressure and shows a gradual increasing trend over time. Although there are certain discrepancies in shrinkage rates among the three cases, the values of Case 2 and Case 3 are relatively close. After 30 years of continuous operation, the total volume shrinkage of An1–An4 and An12 under the three cases is 18.82%, 19.66%, and 19.83%, respectively, all of which are below the 30% threshold and meet the relevant evaluation criteria for stability in terms of volume shrinkage.

It should be noted that the three volume shrinkage rates are close to each other. On the one hand, the fluctuation of internal pressure is insignificant; on the other hand, the salt caverns are large in scale, so the absolute shrinkage volume remains considerable, leading to relatively obvious actual differences.

The volume of the salt cavern is 1,007,972 m^3^. The absolute volume shrinkage under different working conditions is 189,700 m^3^, 198,167 m^3^ and 199,880 m^3^ respectively. The maximum difference among these three values reaches approximately 10,000 m^3^.

In response to the reviewer’s comments, we further analyzed the power law. The displacement variation is correlated with the integral of deviatoric stress over time. In addition, the deviatoric stress values under the three working conditions are relatively close, leading to similar integral results. In addition, interlayers exist within the formation, which can restrain volume shrinkage. Therefore, the above phenomenon is mainly attributed to the similar deviatoric stress and the deformation restraint effect of interlayers.

The volume of salt caverns for CAES power stations is a key indicator determining power generation efficiency. Larger cavern volume contributes to higher power output and better operational efficiency, which is conducive to improving the economic benefit of the power station.

From the comparative analysis of the operating conditions, Case 1 performs well in terms of safety and deformation control. Case 2 maintains structural stability while achieving certain performance improvements by optimizing the upper and lower pressure limits, providing a feasible approach for operational strategy optimization.

## 5. Discussion

Based on the 350 MW salt cavern CAES project in Anning, Kunming, this paper adopts FLAC3D to conduct numerical simulations of static stability and long-term injection–production stability for multi-cavern groups. The evolution mechanisms of the plastic zone, displacement, stress, and volume shrinkage under different internal pressures and long-term cyclic operating conditions are systematically revealed. Most existing studies focus on single-cavern analysis, adopt isotropic assumptions, or only carry out short-term simulations. In this work, the obtained results are comprehensively compared with published mechanical experiments, numerical simulations, and field monitoring data of rock salt. The differences from existing studies are clarified, the underlying mechanical mechanisms are revealed, and the engineering application value of the findings is highlighted.

### 5.1. Comparative Analysis of Transversely Isotropic Mechanical Characteristics of Rock Salt

In this study, when the internal pressure exceeds 4 MPa, the plastic zone is confined to the contact zones between salt rock and interlayers, which is consistent with the mechanical response under the isotropic assumption. Laboratory triaxial tests demonstrate that the tensile and shear strength perpendicular to the bedding plane is lower, leading to a larger plastic zone and higher displacement under the same stress state. The simulation results in this paper are therefore slightly conservative and inherently safe. In practical engineering, interlayers and bedding planes remain the weakest structural positions. It is observed that tensile failure dominates under low internal pressure, and coupled tensile–shear composite failure occurs within interlayers, which is highly consistent with experimental conclusions of transversely isotropic rock salt. Bedding planes tend to act as stress-partitioning interfaces and the initial yield boundaries. The Anning salt formation consists of interbedded rock salt, glauberite, and mudstone. Although the formation is simplified as a homogeneous medium in the simulation, the displacement concentration zones and initial plastic yielding zones coincide well with bedding interfaces. This indicates that even without introducing anisotropic constitutive parameters, the interbedded stratigraphic structure still dominates the overall mechanical response. For refined modeling in future research, transversely isotropic elastic and creep parameters are recommended to further improve the prediction accuracy.

### 5.2. Comparison of the Influence Law of Anisotropy on Long-Term Stability of Salt Caverns

Long-term numerical simulations and physical model tests at home and abroad show that rock salt anisotropy can accelerate creep deformation, expand the range of plastic zones, increase volume shrinkage rate, and change the convergence direction of caverns. Existing studies have proven that anisotropy causes plastic zones to preferentially propagate and penetrate along bedding planes, easily forming zonal yield failure.

In this study, no penetrating plastic zones appeared after 30 years of operation, and plastic deformation was mainly scattered or distributed in bands along interlayers. This is attributed to the relatively high operating internal pressure of 6.0–9.0 MPa adopted in the simulation; the high confining pressure significantly suppresses the weakening effect induced by rock anisotropy. In terms of volume shrinkage, the 30-year shrinkage rates of the three working conditions range from 18.82% to 19.83%, which are lower than most long-term simulation results considering anisotropy. This further demonstrates that high internal pressure operation can effectively offset the adverse effects of anisotropy.

The key consensus is as follows: in layered rock salt, anisotropy does not change the core law of instability under low pressure and stability under high pressure, but only affects the instability rate and local failure morphology. For the Anning project, the cavern layout spacing is reasonable and the operating pressure range is appropriate; hence, anisotropy will not become a dominant controlling risk.

### 5.3. Comparison Between Static Loading and Quasi-Static Cyclic Injection–Production Loading

Current studies divide the stress state of salt caverns into static constant pressure loading and quasi-static cyclic internal pressure loading, which present distinctly different stability mechanisms. Under the same average internal pressure, static constant pressure theoretically provides better stability by avoiding stress fluctuations and reducing the accumulation of fatigue damage. In practical engineering, however, the stability of salt caverns is controlled by the coupled effect of creep and fatigue. Static simulation tends to give optimistic results and is only applicable to preliminary design and boundary condition evaluation, while quasi-static cyclic simulation is more consistent with actual engineering operation characteristics.

The static stability threshold of 4 MPa obtained in this study is consistent with relevant specifications for salt cavern gas storage at home and abroad. The caverns still maintain good stability under long-term cyclic operation, indicating that static analysis results can serve as a reliable safety baseline. The Anning project adopts a daily cyclic operation mode with a high internal pressure range, which avoids risky low-pressure static operation and severe fatigue loading, reflecting a scientific and reasonable operation strategy.

### 5.4. Comprehensive Discussion and Scientific Value

In this paper, the Power-Mohr viscoelastic–plastic creep model is adopted, which can well describe the long-term deformation characteristics of rock salt. Although transversely anisotropic behavior and fatigue damage are not considered, the numerical results are reliable and slightly conservative under the conditions of high internal pressure, gentle interbedded strata and reasonable cavern spacing.

Few existing studies have conducted 30-year cyclic numerical simulation for multi-cavern groups. This study verifies that reasonable cavern spacing combined with a high lower-limit operating pressure can effectively prevent plastic penetration, stress superposition and deformation coupling among multiple caverns, providing a direct reference for the design of large-capacity multi-cavern CAES projects in China.

Comparative analysis shows that Case 1 achieves the best stability; Case 2 balances structural safety and energy storage efficiency; and Case 3 requires enhanced monitoring of local high-stress regions. The above conclusions are consistent with the international design and operation concept of salt caverns: high-pressure operation, narrow pressure range and minor pressure fluctuation.

The Anning salt formation features moderate salt grade, appropriate burial depth and stable stratigraphic structure. The multi-field index evaluation system established in this paper can be directly applied to similar layered rock salt CAES projects in Southwest China.

## 6. Conclusions

Taking the 350 MW CAES demonstration project in Anning, Kunming as the research background, this paper establishes a three-dimensional numerical model using FLAC3D to systematically evaluate the stability of multi-cavern salt cavern gas storage clusters under static loading and long-term cyclic injection–production conditions. The evolution characteristics of plastic zone distribution, surrounding rock displacement, stress field response and cavern volume shrinkage under different operational scenarios are comprehensively analyzed, and practical optimization recommendations for engineering operation parameters are further proposed. The main conclusions are summarized as follows:

(1) The static stability evaluation indicates that the internal pressure level exerts a significant control on the development of plastic zones in the surrounding rock of the caverns. At high internal pressure (4–9.5 MPa), plastic zones are mainly confined to interlayer contact zones, the surrounding rock is dominated by compressive stress, and the overall structure is stable with good integrity. When the internal pressure drops below 4 MPa, the scope of plastic zones expands significantly. Tensile failure dominates in the salt rock layer, while tension–shear composite failure mostly occurs in interlayers. Local tensile stress concentrates at interlayer boundaries and cavern corners, indicating potential risks of stress reversal and layered instability. The surrounding rock deformation of the caverns is characterized by bottom heave and roof subsidence. The maximum surface displacement reaches approximately 11.5 cm, with no obvious differential settlement observed, indicating that the overall ground deformation remains well controllable.
(2) The long-term stability evaluation reveals that under the three injection–production conditions, all caverns follow the common law that “plastic zones expand gradually with operation time, displacements accumulate continuously, and stresses stabilize after initial accumulation”. After 30 years of operation, no through-going plastic zones appear in any cavern, the deformation range is limited and develops slowly, and no through-going failure structure is formed. The principal stresses do not exceed the upper limit of tensile strength of rock salt, and the volume shrinkage rates are all lower than 30%, meeting the relevant stability evaluation criteria. This indicates that the cavern spacing is reasonably arranged, the cavern structure is safe and controllable for long-term operation, and there is good engineering feasibility.
(3) The comparison of different injection–production conditions shows that Case 1 performs the best in safety and deformation control. Case 2 improves operation efficiency on the premise of ensuring stability by optimizing the operating pressure range, which can provide a feasible scheme for practical engineering operation. Although Case 3 maintains overall stability, it exhibits delayed stress release and prominent local high-stress concentrations. Therefore, enhanced long-term in situ stress monitoring is recommended during actual operation.

## 7. Limitations of This Study

This study still has several limitations that need to be improved in further research. Firstly, the established geological and cavern model has properly simplified the actual stratum and interlayer structure, ignoring the local complex geological variations and micro-defect distribution of rock masses. Secondly, uniform mechanical and creep parameters are adopted for salt rock and mudstone in the simulation, without considering the spatial heterogeneity of rock mass parameters and the long-term time-varying degradation effect under hydro-mechanical coupling. Thirdly, only three typical periodic injection–production schemes are designed in this paper, while the influences of the random fluctuation of renewable energy output and the actual variable operating load are not fully reflected. Moreover, this research mainly concentrates on the mechanical stability of surrounding rock, and the multi-field coupling effects of seepage, thermodynamics and gas tightness inside the salt cavern are not involved.

In future work, more refined geological models, spatially varied rock parameters and actual fluctuating operation modes will be adopted, and multi-field coupling simulation will be conducted to further improve the accuracy and applicability of stability evaluation for salt cavern CAES.

## Figures and Tables

**Table 3:** Mechanical parameters of constitutive model.

Lithology	Elastic Modulus/GPa	Poisson’s Ratio	Cohesion/MPa	Angle of Internal Friction/°	Tensile Strength/MPa	A/MPa^−*n*^∙h^−1^	*n*
Saline rock	3.66	0.28	11.04	41.34	1.29	8.89 × 10^−7^	2.618
Mudstone	11.56	0.26	11.45	34.44	3.66	2.30 × 10^−8^	2.0

## Data Availability

The original contributions presented in this study are included in the article. Further inquiries can be directed to the corresponding author.
